# Grand Challenge at the Frontiers of Synaptic Neuroscience

**DOI:** 10.3389/fnsyn.2021.748937

**Published:** 2021-10-20

**Authors:** P. Jesper Sjöström

**Affiliations:** Department of Medicine, Department of Neurology and Neurosurgery, Centre for Research in Neuroscience, The Research Institute of the McGill University Health Centre, Montreal General Hospital, Montreal, QC, Canada

**Keywords:** synapse, plasticity, learning, memory, methods, techniques, pathology

## Introduction

The brain does not operate like a typical computer—there is no central processing unit, there are no separate memory banks, and performance degrades gracefully when individual components fail. This is because all brain functioning is distributed across vast networks of neurons, which are connected by synapses that process and store information. To understand the brain, we therefore need to know how synapses work, hence the central importance of the Synaptic Neuroscience research field that this Frontiers Specialty Journal covers.

In 2000, Abbott and Nelson overviewed the field of synaptic plasticity and likened it to the taming of a beast (Abbott and Nelson, 2000). Synaptic plasticity is indeed a particularly daunting task in neuroscience: experiments are slow and challenging, outcomes vary with precise experimental conditions, and interpretations of the results are inherently prone to disagreement, due to experimental as well as theoretical complexities. We are now more than two decades after the publication of Abbott and Nelson's review, we have seen the immense progress since, and we are in a good position to speculate on what is at the Frontiers of Synaptic Neuroscience. Here, in personal view, I have collected a non-exhaustive list of challenges, phrased as questions.

## How Does One Build a Synapse?

The key building blocks of the active zone (Südhof, 2012) and the postsynaptic density (Sheng and Kim, 2011) of central synapses are well described. Even so, we may have only barely scratched the surface. Particularly intriguing are the scenarios where the target cell type determines presynaptic release mechanisms, such as in the case of Elfn1 (Stachniak et al., 2019), which—given the diversity of cell types in the brain—implies a potentially enormous diversity of synaptic building blocks across different synapse types. This question is furthermore tightly linked to a related question: How are synapse built during development?

## How Can We Map Plasticity at All Synapse Types?

It has long been known that short-term plasticity at connections made by the same axon can vary dramatically depending on the target neuron type (e.g., Markram et al., 1998). Since the postsynaptic cell determines presynaptic release, this is really a matter of synapse-type rather than cell-type specificity (Blackman et al., 2013). This synapse-type specificity also holds true for long-term plasticity—both phenomenology and mechanisms can vary dramatically depending on the form of synapse at hand (Toth and McBain, 2000; Larsen and Sjöström, 2015). Since there is a plethora of cell types in the brain, there may therefore be a corresponding multiplicity of synapse-type-specific learning rules. Today, the state-of-the-art approach for achieving synapse-type-specific experiments—where both the pre- and the postsynaptic cell identities are known—is still the paired-recording technique. This technique, however, is painstaking and challenging. To create a relatively complete library of synaptic learning rules across all brain regions—*the plasticitome*, the learning rule equivalent to the genome or the connectome—we clearly need novel approaches that have ten to a hundred-fold higher throughput than the paired recording technique. Solving the plasticitome will probably require combining multiple techniques, including electrode arrays (Field et al., 2020), multiple simultaneous whole-cell recordings (Perin and Markram, 2013; Lalanne et al., 2016), patch robots (Annecchino et al., 2017; Suk et al., 2017), and optogenetics (Zhang and Oertner, 2007; Emiliani et al., 2015).

## Is There a Grand Unifying Theory of Synaptic Learning Rules?

Many studies have tried to establish if calcium alone can account for synaptic plasticity (Shouval et al., 2002; Rackham et al., 2010; Chindemi et al., 2020), but given the involvement of multiple mechanisms based on dendrite biophysics (Froemke et al., 2010), presynaptic NMDA receptors (Wong et al., 2021) or NMDA receptors that signal unconventionally (Dore et al., 2017), metabotropic glutamate receptors (Nevian and Sakmann, 2006), and learning mechanisms residing in a third cell such as astrocytes (Min and Nevian, 2012; Adamsky et al., 2018), can we really hope to find one calcium model that fits all synapse types? If the calcium hypothesis in synaptic plasticity is not valid for all synapse types, then can we at least establish a phenomenological model that captures plasticity at all synapses? Perhaps it is possible to derive a phenomenological model that can be easily tweaked to account for factors such as synapse position in the dendritic arbor (e.g., Clopath and Gerstner, 2010; Clopath et al., 2010), but whether such an approach is *generally* feasible for all synapse and cell types remains unknown. Clearly, plasticity at some synapse types does not conform to classical NMDA receptor dependent plasticity, e.g., parallel fibers onto cerebellar Purkinje cells (Hansel et al., 2001), or mossy fibers onto hippocampal CA3 pyramidal cells (Zalutsky and Nicoll, 1990). Also, in a contrasting view to the Grand Unifying Theory, Suvrathan (2018) has convincingly argued that learning rules may in fact be intrinsically heterogenous, to form a basis for learning the behavioral tasks at hand. But perhaps it will be possible to establish a small set of canonical models for a handful of archetypical central synapse classes? Such an effort would help typify synapses of the brain, eventually leading to a robust synapse classification scheme.

## How Does the Brain Solve the Credit Assignment Problem?

Artificial neural networks have in recent years outperformed humans in several complex tasks. Key to this success is deep learning, i.e., the use of multi-layered networks in combination with the error backpropagation algorithm. This algorithm is used during a separate training phase to assign credit to each synapse, so that the network output error travels backwards to tweak synaptic strengths individually. The brain is clearly a very deep network, so how does it solve the credit assignment problem? Presumably, information cannot travel backwards across synapses in the brain. Storing and using information are furthermore not separate modes of operation for the real brain; your brain is online and functional as you learn new things.

Recent theoretical studies have offered exciting new solutions to these problems. For example, Payeur et al. (2021) recently proposed that by combining short-term plasticity, local dendritic regenerative events, and long-term plasticity in feedback pathways, deep learning can be effectively mimicked by spike burst-dependent plasticity. However, most theoretical propositions similar to this one remain to be validated experimentally (Richards and Lillicrap, 2018; Richards et al., 2019), which can be technically quite challenging.

Because of issues such as the credit assignment problem, it is not clear that the classical forms of cellular learning that dominate the present literature—e.g., long-term potentiation (LTP; Malenka and Bear, 2004) and spike-timing-dependent plasticity (STDP; Markram et al., 2012)—are enough to account for information storage in the brain. As a consequence, demonstrating how the brain solves the credit assignment problem may also tell us which type of synaptic learning rules discovered *in vitro* are actually relevant in the intact brain, which has been a long-standing debate (Lisman and Spruston, 2005, 2010).

Having said that, it is also important to remember that the question “*How does the brain solve the credit assignment problem?*” is potentially misleading, because the answer might be: it doesn't. The best biological and technological solutions need not be identical; planes do not flap their wings like birds do.

## Does Long-Term Synaptic Plasticity Underlie Learning and Memory?

In a short review, Stevens (1998) asked what he called the million-dollar question: *Does LTP* = *memory?* He outlined four essential experiments that should be carried out to demonstrate that LTP underlies behavioral learning and memory: blocking LTP prevents behavioral learning; synapses exhibit LTP; behavioral learning causes LTP that when blocked abolishes the learning; and inducing LTP causes behavioral learning. Although it has been established that *LTP* = *memory* in simpler model systems—such as for the gill withdrawal reflex in the sea slug Aplysia (Kandel, 2001)—with the Stevens (1998) definition, it actually remains to be demonstrated that *LTP* = *memory* in most mammalian learning models. In fact, most studies of long-term plasticity do not explore beyond an hour or two; clearly not enough to establish a direct link with long-term memory formation (Stevens, 1998). Perhaps the closest we currently get to satisfying the four requirements of Stevens (1998) in a single paper can be found in the amygdala fear conditioning study by Nabavi et al. (2014). It may, however, take some time before the same can be achieved for brain regions with more complex roles in learning, such as the hippocampus (Tonegawa et al., 2018).

## What is the Synaptic Basis of Neuropathology?

Malfunction of synapses and of their plasticity can contribute to severe neuropathology, which has led to the notion of a possible synaptic basis of disease (Lüscher and Isaac, 2009). Epilepsy is a prime example of how excessive activity can hijack plasticity in the healthy brain to yield pathology (Cela and Sjöström, 2019). In fact, both the postsynapse (Kasai et al., 2021) and the presynapse (Van Battum et al., 2015; Perrone-Capano et al., 2021) have been shown to contribute to a broad range of brain diseases as diverse as schizophrenia, autism spectrum disorder, Alzheimer's disease, and amyotrophic lateral sclerosis. By considering neuropathologies as synaptic disease, we may increase our chances of finding novel and specific therapies, since different synapse types are often mechanistically distinct. For example, Walia et al. (2021) recently demonstrated that by specific pharmacologic blockade of calcium-permeable but not calcium-impermeable AMPA receptors in the cochlea, it was possible to protect from excitotoxic pathology during acoustic overexposure, without impairing hearing as such. Promising new treatments will thus likely require detailed mechanistic knowledge of the synaptic pathways at hand.

## What Will the Next Big Technological Breakthroughs Be?

There is a broad consensus that major advances in neuroscience are typically driven by new technologies (Bassett et al., 2020). It is for example difficult to underestimate the impact of optogenetics (Boyden et al., 2005) on modern neuroscience. Key to the next big breakthroughs will be the audacity to ask daring and wishful questions, such as: How can we read out all the synaptic weights in live brain tissue? Because what was once science fiction—e.g., the activation of individual neurons with light—is now science.

## Author Contributions

The author confirms being the sole contributor of this work and has approved it for publication.

## Funding

PJS was funded by FRQS *Chercheur-Boursier* award 254033.

## Conflict of Interest

The author declares that the research was conducted in the absence of any commercial or financial relationships that could be construed as a potential conflict of interest.

## Publisher's Note

All claims expressed in this article are solely those of the authors and do not necessarily represent those of their affiliated organizations, or those of the publisher, the editors and the reviewers. Any product that may be evaluated in this article, or claim that may be made by its manufacturer, is not guaranteed or endorsed by the publisher.
